# Body mass index and blood volume influence plasma biomarkers and positron emission tomography classification in preclinical Alzheimer's disease

**DOI:** 10.1002/alz.70765

**Published:** 2025-10-11

**Authors:** Tovia Jacobs, Courtney O’ Brien, Luisa Figueredo, Alexandra Gogola, Naomi L. Gaggi, Brian Hurwitz, Elizabeth Pirraglia, Shimon Herzog, Jaime Ramos‐Cejudo, Timothy M. Shepherd, Priya Palta, Juan Fortea, Thomas M Wisniewski, Rebecca A. Betensky, Brian Lopresti, Michelle M. Mielke, Antonio Convit, Ricardo S. Osorio, for the Alzheimer's Disease Neuroimaging Initiative

**Affiliations:** ^1^ Department of Psychiatry New York University Grossman School of Medicine New York USA; ^2^ Penn State College of Medicine Hershey Pennsylvania USA; ^3^ Department of Radiology University of Pittsburgh School of Medicine Pittsburgh Pennsylvania USA; ^4^ VA Boston Cooperative Studies Program MAVERIC VA Boston Healthcare System Boston Massachusetts USA; ^5^ Department of Radiology New York University Grossman School of Medicine New York USA; ^6^ Department of Neurology University of North Carolina North Carolina USA; ^7^ Sant Pau Memory Unit Department of Neurology Institut d'Investigacions Biomèdiques Sant Pau Hospital de Sant Pau Universitat Autònoma de Barcelona Barcelona Spain; ^8^ Barcelona Down Medical Center Fundació Catalana Síndrome de Down Barcelona Spain; ^9^ Centro de Investigación Biomédica en Red en Enfermedades Neurodegenerativas (CIBERNED) Instituto de Salud Carlos III (CIBER‐ISCIII) Madrid Spain; ^10^ Center for Cognitive Neurology Department of Neurology New York University Grossman School of Medicine New York USA; ^11^ Department of Pathology New York University Grossman School of Medicine New York USA; ^12^ Department of Biostatistics NYU School of Global Public Health 708 Broadway New York USA; ^13^ Department of Epidemiology and Prevention Wake Forest University School of Medicine Winston‐Salem North Carolina USA; ^14^ Nathan Kline Institute Orangeburg New York USA

**Keywords:** Alzheimer's disease, amyloid, blood volume, blood‐based biomarkers, body mass index, dilution, glial fibrillary acidic protein, neurodegeneration, neurofilament light, obesity, p‐Tau181, p‐Tau217, PET, plasma, tau

## Abstract

**INTRODUCTION:**

Blood‐based biomarkers (BBMs) are promising tools for Alzheimer's disease (AD) diagnosis, but their accuracy may be affected by body mass index (BMI) and blood volume (BV) through dilution. We investigated how BMI and BV influence BBM concentrations and PET prediction.

**METHODS:**

Data from 241 cognitively unimpaired participants in the Alzheimer's Disease Neuroimaging Initiative (ADNI) were examined to evaluate the influence of BMI/BV on BBMs (Aβ_42/40_, p‐Tau_181_, p‐Tau_217_, glial fibrillary acidic protein [GFAP], neurofilament light chain [NfL]) and BBM‐based PET predictions.

**RESULTS:**

Elevated BMI/BV associated with lower BBM concentrations, especially for p‐Tau_217_ and NfL, independent of brain amyloid burden. BMI‐stratified thresholds improved amyloid PET prediction, with higher BBM thresholds and area under the curve (AUC) values seen in normal weight compared to overweight or obese participants. Drastic BMI/BV declines due to weight loss increased BBM variability and systematic PET misclassification.

**DISCUSSION:**

Adjusting for BMI/BV in BBM‐based diagnostics appears to improve accuracy and reliable detection of AD pathology, especially in preclinical stages.

**Highlights:**

- Body mass index (BMI) and blood volume (BV) significantly influenced plasma BBM concentrations in cognitively unimpaired (CU) individuals.
- Blood‐based biomarkers (BBMs) associated more strongly with BV than with BMI.
- Dilution effects were independent of brain amyloid burden.
- BMI‐stratified BBM thresholds improved amyloid positron emission tomography (PET) classification accuracy.
- Declines in BMI/BV resulted in PET prediction bias and systematic errors.

## 1 BACKGROUND

Alzheimer's disease (AD), the leading cause of dementia, is characterized by the accumulation of amyloid beta (Aβ) plaques, neurofibrillary tangles, and neurodegeneration.^1^ To standardize AD clinical research efforts, Jack et al. proposed the amyloid/tau/neurodegeneration (ATN) framework as a biomarker‐based system for classifying AD pathology and staging disease progression.^2^ This framework was later expanded to incorporate inflammation, vascular dysfunction, and α‐synucleinopathy (IVS) as additional components relevant to AD‐neurodegeneration.^3^ The gold standard models for ATN classification include positron emission tomography (PET) and cerebrospinal fluid (CSF) biomarker assays, with magnetic resonance imaging (MRI) playing a supportive role.^4^ However, the limited availability and high cost of PET tracers and the invasiveness of lumbar punctures spurred a shift toward the development of blood‐based biomarkers (BBMs).^3^, ^5^, ^6^ Emerging evidence suggests that AD BBMs can effectively identify PET‐classified amyloid positivity across the clinical spectrum.^7^, ^8^, ^9^, ^10^, ^11^, ^12^, ^13^, ^14^ Promising BBMs are Aβ peptides (Aβ_40_ and Aβ_42_), phosphorylated tau (p‐Tau_181_, p‐Tau_217_, and p‐Tau_231_), neurofilament light (NfL), and glial fibrillary acidic protein (GFAP).^7^, ^8^, ^9^, ^10^, ^11^, ^12^, ^13^, ^15^, ^16^

As BBMs become integral to AD diagnosis, it is essential to consider physiological factors that may influence their concentration and interpretation. Obesity has been implicated in both AD risk and biomarker variability, with effects that differ across the lifespan. Midlife obesity is associated with an increased risk of AD and dementia, whereas weight loss in late life has been indicated as a noncognitive sign of AD, suggesting distinct mechanisms at different stages of aging and disease progression.^17^, ^18^, ^19^, ^20^, ^21^, ^22^, ^23^ Unsurprisingly, BMI is known to be associated with AD BBMs.^12^, ^24^, ^25^, ^26^, ^27^, ^28^, ^29^, ^30^, ^31^ However, emerging evidence suggests that these associations may, in part, be driven by blood volume (BV) effects rather than direct associations with AD pathology.^24^, ^26^, ^27^, ^30^ BV, comprised of plasma and cellular components, represents the total volume of blood circulating within an individual and is proportional to body size.^32^ Assuming BBMs reflect AD pathology, their concentration in blood may depend not only on the extent of underlying pathology but also on the volume in which they are distributed. In individuals with higher BV, such as those with obesity, BBM concentrations may appear artificially lower due to dilution, despite comparable pathological burden. Conversely, in individuals with lower BV, such as those who are dehydrated, underweight, or experience age‐ or AD‐related weight loss, BBM concentrations may appear artificially elevated. Therefore, BV may either amplify or obscure pathological signals in BMI‐BBM associations depending on aging and disease status. If AD BBMs are influenced in part by BMI through BV‐related dilution, rather than solely reflecting AD pathology, current BBM cutoffs for predicting amyloid PET positivity may need to be stratified by BMI category.

While prior studies linking BBMs with amyloid PET have adjusted for BMI, they have primarily modeled it as a static covariate, overlooking potential interactive effects between BMI and BBMs on PET outcomes, as well as longitudinal changes in BMI with aging, AD progression, and accompanying shifts in BV. Additionally, prior studies have often focused on individuals with mild cognitive impairment (MCI) or dementia, groups with large variability of disease burden, making it difficult to isolate the effects of BMI from coexisting neurodegenerative and systemic comorbidities. However, the issue of blood dilution might be particularly important in preclinical stages, when BBM concentrations more frequently hover near positivity thresholds and subtle fluctuations could be attributed to either disease progression or BV‐related dilution effects.

To address these considerations, we used data from the Alzheimer's Disease Neuroimaging Initiative (ADNI) to examine how BMI and BV relate to AD BBMs (Aβ_42/40_, p‐Tau_181_, p‐Tau_217_, GFAP, and NfL) in cognitively unimpaired (CU) individuals. We tested whether BMI‐ and BV‐related dilution effects independently influence BBM levels, both cross‐sectionally and in relation to changes over time, and we investigated how these factors might affect amyloid PET prediction. Our aim was to determine if current BBM‐based PET cutoffs require adjustment to more accurately capture emerging AD pathology in preclinical stages.

## 2 METHODS

### 2.1 Participant data

All participants included in this study were enrolled in the ADNI. ADNI is an ongoing study that was launched in 2003 as a public–private partnership, led by principal investigator, Michael W. Weiner, MD. The primary focus of ADNI has been to test the clinical progression of AD through a longitudinal study cohort while collecting clinical, biochemical, genetic, and imaging data. ADNI participants were recruited between the ages of 55 and 90 years with cognitive status defined as CU, MCI, and AD. ADNI is a multisite study that abides by a standard protocol, and each site involved in data collection received local institutional review board (IRB) approval. Written informed consent was obtained from enrolled participants.

**RESEARCH IN CONTEXT**

1. **Systematic review**: We reviewed literature on body mass index/blood volume (BMI/BV) influences on Alzheimer's disease (AD) blood‐based biomarkers (BBMs) and BBM‐positron emission tomography (PET) associations. Prior studies link higher BMI to reduced BBMs (e.g., p‐Tau217, neurofilament light chain [NfL]) but treat BMI as static covariates in symptomatic cohorts. BV dilution has been proposed, yet no work has tested BMI/BV as modifiers of BBM‐PET associations in cognitively unimpaired individuals.
2. **Interpretation**: We found BMI and BV significantly modulate BBM‐PET associations through dilution mechanisms, with stronger BV effects. BMI/BV‐specific thresholds improved PET classification and reduced misclassification. Longitudinal BMI/BV decreases associated with BBM increases independent of amyloid burden, consistent with dilution.
3. **Future directions**: Studies should integrate precise BV measures and diverse cohorts using multimodal imaging to clarify dilution‐disease interactions. Targeted investigations of BBM reliability during weight loss from aging, lifestyle factors, and GLP‐1‐GIP receptor agonists therapies are essential for optimizing BBM‐based preclinical AD diagnostics, especially for individuals near positivity thresholds.

In this analysis, only participants who were CU through the duration of the analysis were included to prevent confounding from AD‐related factors such as neurodegenerative processes, co‐pathologies, and associated weight loss, which might further influence biomarker concentrations and mask the influence of dilution effects. Further, the present study focused only on participants with available AD plasma biomarker data as part of a sub study conducted by the Foundation for the National Institutes of Health (FNIH).^33^ To ensure temporal alignment across assessments, the time between blood collection, PET imaging, cognitive evaluation, and physical exam visits was precisely calculated based on exam dates recorded at each visit, rather than relying solely on visit number. Updated study information for all ADNI waves is available on the ADNI website, and all ADNI data included in this study were obtained directly from the study website.^34^

### 2.2 BBM quantification

BBM measures were sourced from the dataset published by the FNIH Biomarkers Consortium which compared seven leading BBM assay platforms from Quanterix Accelerator Laboratory, C2N Diagnostics, National Centralized Repository for Alzheimer's Disease Biomarker Assay Laboratory (NCRAD‐BAL), and the University of Gothenburg.^33^ Separate plasma aliquots were analyzed for each assay platform. Plasma samples were collected, transported, and stored according to ADNI and FNIH Biomarkers Consortium protocols described on the ADNI website.^34^

Specific BBMs measured by these assays included Aβ_40_, Aβ_42_, Aβ_42/40_ ratio, p‐Tau_181_, p‐Tau_217_, nonphosphorylated tau at threonine 217 (*n*‐pTau_217_), the p‐Tau_217_/n‐pTau_217_ ratio, GFAP, and NfL. The number of participants successfully analyzed varied by platform and is detailed in Table S1, along with corresponding assay details. Plasma concentrations for selected BBMs were log10‐transformed before analysis to account for skewness and kurtosis (Table S1). Quanterix assays were run in duplicate, and samples with a coefficient of variation (CV) > 25% were excluded from analysis (*n* = 10; *n* = 6 for p‐Tau_217_ [Janssen], *n* = 3 for p‐Tau_217_ [Alzpath], *n* = 1 for Aβ_42_).

### 2.3 Amyloid PET

Amyloid PET values were sourced from the dataset published on the ADNI website by the Helen Wills Neuroscience Institute at UC Berkeley. For the present study, [18F]‐florbetapir (FBP) was the tracer used to quantify cortical summary standard uptake value ratios (SUVRs). For cross‐sectional analyses, including receiver operating characteristic (ROC) curves, SUVRs were intensity normalized by the whole cerebellum and amyloid positivity was categorized using a threshold of 1.11 SUVR. For longitudinal analyses, SUVRs were intensity normalized by composite reference regions, and amyloid positivity was categorized by a threshold of 0.78 SUVR. This difference in SUVR cut points reflects differences in reference region normalization rather than discrepancies in amyloid burden. The whole cerebellum reference is preferred for cross‐sectional studies due to its stability across individuals, whereas the composite reference region reduces noise and scan‐to‐scan variability, making it more suitable for longitudinal analyses. The 0.78 SUVR threshold was derived through linear transformation of the whole cerebellum‐normalized threshold to maintain consistency in amyloid classification over time. These thresholds were pre‐determined and suggested by the authors at UC Berkeley. More information on these methods can be found by downloading the UC Berkeley—Amyloid PET Processing Methods document, which requires ADNI access.

Each FBP‐SUVR threshold can be converted to the centiloid scale using methods described previously.^35^ Specifically, the 1.11 SUVR threshold (whole cerebellum normalization) corresponds to approximately 19.8 Centiloids (CL = 188.22×SUVR‐189.16), while the 0.78 SUVR threshold (composite reference normalization) corresponds to approximately 25.7 CL (CL = 300.66×SUVR‐208.84).^35^, ^36^ However, because FBP was the sole tracer used in this analysis, normalized SUVR values were retained as outcomes to preserve measurement accuracy.

### 2.4 Cognitive status

Cognitive diagnostic criteria were evaluated using the DXSUM.csv file downloaded directly from the ADNI website on 02/04/2025. Included participants received a clinical diagnosis of CU (DIAGNOSIS = 1 in the DXSUM file) within 6 months of plasma collection, or at a follow‐up visit. A number of participants (*n *= 30) showed reversion from MCI to CU, in which case they were considered CU for the present study.

### 2.5 BMI and BV calculations

Height and weight data used to calculate BMI and BV variables were obtained from the VITALS.csv file downloaded directly from the ADNI website on 02/04/2025. For participants with missing height at a given visit, height was imputed using the value from the previous visit or the average of the preceding and following visits. To assess potential bias from interpolated height values, we modeled height as a function of age, using all visits where height was recorded for included participants (*n* = 304). Linear mixed models revealed a significant but modest decline of 0.20 ± 0.04 cm in height per year of aging (*p* < 0.001). Considering the average time between height measurement and BMI/BV calculation in our sample was 2.05 years [Q1 = 0.096, Q3 = 3.57], interpolation likely introduced minimal bias. In contrast to height, weight was treated more strictly due to its potential to fluctuate over short periods. Any visit with missing weight was excluded (*n* = 13), and no imputations were performed, ensuring valid temporal alignment between weight and BBM measurements.

1. BMI

BMI was calculated using the formula:

$$BMI=\frac{weight\left(kg\right)}{height{\left(m\right)}^{2}}$$

2. BV

BV was calculated using the Nadler equation, which differs by sex:^32^

For males:

$$0.3669\times height{\left(m\right)}^{3}+0.03219\times weight\left(kg\right)+0.6041$$

For females:

$$0.3561\times height{\left(m\right)}^{3}+0.03308\times weight\left(kg\right)+0.1833$$

Although the Nadler equation differs by sex, we retained sex as a covariate in adjusted regression models to account for biological differences beyond BV. To evaluate potential multicollinearity, we calculated variance inflation factors (VIFs) for models including both BV and sex across all BBMs. Across all models, the VIFs for BV and sex were consistently low (∼2.2–2.4) and below conventional thresholds for multicollinearity concern (VIF > 5).

### 2.6 Statistical analysis

All statistical analyses were conducted using R Version 4.2.2. The *tableone* package^37^ was used to generate Table 1, comparing cohort characteristics via Pearson's chi‐squared test for categorical variables and *t*‐tests for continuous variables. For binary proportion estimates, 95% confidence intervals were calculated using the Wilson score method. All linear mixed‐effects models (LMMs) were generated using the *lmerTest* package^38^ and contained random intercepts to account for within‐subject correlation across multiple visits. All ROC curve analyses were conducted using the *pROC* package.^39^ Matching procedures were conducted using the *MatchIt* package,^40^ and the *ggplot2* package^41^ was used for data visualizations. In this study, *p* values ≤ 0.05 were considered significant, while *p* values ≤ 0.10 were considered marginally significant.

**TABLE 1:** Cohort characteristics at baseline.

		By BMI group			
Parameter	Full cohort	Normal weight	Overweight	Obese	*p‐*value
N	241	71	113	56	
Age, mean (SD)	73.4 (6.8)	74.5 (7.2)	73.7 (6.8)	71.5 (6.2)	**0.042** ^*^
Sex = male (%)	111 (46.1)	30 (42.3)	61 (54.0)	20 (35.7)	0.059
Race (%)					0.742
White	221 (91.7)	66 (93.0)	102 (90.3)	53 (94.6)	
Black or African American	12 (5.0)	2 (2.8)	8 (7.1)	2 (3.6)	
Asian	3 (1.2)	1 (1.4)	1 (0.9)	0 (0.0)	
American Indian or Alaskan Native	1 (0.4)	1 (1.4)	0 (0.0)	0 (0.0)	
More than one race	4 (1.7)	1 (1.4)	2 (1.8)	1 (1.8)	
Education, years, mean (SD)	16.62 (2.52)	16.86 (2.53)	16.58 (2.54)	16.34 (2.49)	0.508
*APOE* ε4 = carrier (%)	72 (29.9)	24 (33.8)	35 (31.0)	13 (23.2)	0.413
Height, cm, mean (SD)	168.06 (9.84)	167.41 (10.09)	169.05 (9.66)	166.98 (9.95)	0.348
Weight, kg, mean (SD)	77.82 (15.74)	63.82 (9.58)	78.71 (9.37)	94.29 (15.47)	**<0.001** ^***^
BMI, mean (SD)	27.49 (4.83)	22.67 (1.67)	27.48 (1.46)	33.79 (4.68)	**<0.001** ^***^
BV, mean (SD)	4.65 (0.86)	4.16 (0.77)	4.74 (0.76)	5.12 (0.86)	**<0.001** ^***^
Aβ_42/40_^(C2N)^, mean (SD)	0.09 (0.01)	0.10 (0.01)	0.09 (0.01)	0.09 (0.01)	0.175
Aβ_42/40_^(Fujirebio)^, mean (SD)	0.09 (0.02)	0.09 (0.01)	0.09 (0.02)	0.09 (0.01)	0.553
Aβ_42/40_^(Roche)^, mean (SD)	124.48 (17.82)	124.01 (19.84)	123.31 (15.56)	127.05 (19.16)	0.452
Aβ_42/40_^(Quanterix)^, mean (SD)	0.06 (0.01)	0.06 (0.01)	0.06 (0.01)	0.06 (0.01)	0.539
p‐Tau_181_^(Roche)^, mean (SD)	1.02 (0.50)	1.06 (0.50)	1.03 (0.56)	0.94 (0.35)	0.396
p‐Tau_181_^(Quanterix)^, mean (SD)	19.55 (13.10)	20.30 (11.91)	18.84 (9.77)	20.21 (19.08)	0.743
p‐Tau_217_^(C2N)^, mean (SD)	1.88 (2.01)	2.31 (2.32)	1.86 (2.05)	1.37 (1.29)	**0.036** ^*^
p‐Tau_217_/N‐ptau_217_^(C2N)^, mean (SD)	3.49 (2.96)	4.50 (3.76)	3.29 (2.56)	2.61 (2.00)	**0.001** ^***^
p‐Tau_217_^(Fujirebio)^, mean (SD)	0.16 (0.24)	0.20 (0.26)	0.17 (0.27)	0.10 (0.08)	0.069^†^
p‐Tau_217_^(Quanterix,Alzpath)^, mean (SD)	0.37 (0.26)	0.40 (0.27)	0.38 (0.29)	0.30 (0.17)	0.103
p‐Tau_217_^(Quanterix,Janssen)^, mean (SD)	0.05 (0.04)	0.06 (0.04)	0.05 (0.04)	0.04 (0.03)	0.083^†^
GFAP^(Roche)^, mean (SD)	0.10 (0.06)	0.11 (0.05)	0.10 (0.06)	0.09 (0.06)	0.29
GFAP^(Quanterix)^, mean (SD)	144.97 (78.76)	153.52 (61.84)	141.04 (86.30)	135.62 (68.18)	0.456
NfL^(Roche)^, mean (SD)	3.52 (1.46)	4.02 (1.49)	3.34 (1.32)	3.12 (1.39)	**0.001** ^***^
NfL^(Quanterix)^, mean (SD)	19.22 (8.83)	22.24 (8.61)	17.81 (8.52)	17.81 (8.18)	**0.005** ^**^
Days from height/weight visit to plasma visit, mean (SD)	4.00 (6.40)	3.93 (6.05)	3.79 (6.95)	3.82 (6.30)	0.990
Amyloid PET summary SUVR, mean (SD)	1.10 (0.20)	1.13 (0.22)	1.11 (0.21)	1.06 (0.15)	0.175
Amyloid PET, Centiloids, mean (SD)	18.49 (37.16)	22.51 (40.79)	20.04 (38.61)	10.73 (27.77)	0.178
Amyloid PET positivity (%)	75 (31.1)	25 (35.2)	38 (33.6)	12 (21.4)	0.189
Days from PET to plasma visit, mean (SD)	10.76 (15.81)	−2.97 (13.61)	−7.77 (20.51)	−6.79 (17.97)	0.209
Longitudinal data					
*N*, participants with longitudinal data	211 (87.6)	57 (80.3)	99 (87.6)	54 (96.4)	**0.024** ^*^
No. of visits, mean (SD)	2.75 (0.48)	2.65 (0.48)	2.79 (0.48)	2.78 (0.46)	0.188
Years followed, mean (SD)	5.23 (2.11)	4.84 (2.32)	4.8 (2.6)	5.46 (1.99)	0.211

*Note*: Differences in categorical variables were assessed using chi‐squared tests, and differences in continuous variables were assessed using one‐way ANOVA, conducted through the *tableone* package in R. *APOE* ε4 positivity was defined as the presence of at least one *APOE* ε4allele. Significance levels are denoted as: ^†^ *p* ≤ 0.100; ^*^ *p* ≤ 0.050; ^**^ *p* ≤ 0.010; ^***^ *p* ≤ 0.001.

Abbreviations: Aβ, amyloid β; ANOVA, analysis of variance; APOE, apolipoprotein E; BMI, body mass index; BV, blood volume; GFAP, glial fibrillary acidic protein; NfL, neurofilament light; N‐ptau, non‐phosphorylated tau; PET, positron emission tomography; p‐Tau, phosphorylated tau; SD, standard deviation; SUVR, standard uptake value ratio.

#### 2.6.1 Continuous associations between BMI/BV and BBMs

We used LMMs to examine associations between BMI or BV and BBMs encompassing all participant visits. Each BBM was modeled separately, with the BBM as the dependent variable, either BMI or BV as the primary exposure, and age, sex, race, education, apolipoprotein E (*APOE)* ε4, and amyloid PET SUVR included as covariates. Random intercepts were incorporated to account for within‐subject correlation across visits. BMI and BV were z‐score standardized by centering each variable at its mean and scaling by its standard deviation to enable direct comparison of β coefficients.

#### 2.6.2 Associations between BBMs and amyloid PET SUVR

Next, we used LMMs to investigate direct associations between BBMs and amyloid PET SUVR, encompassing all participant visits. Each BBM was modeled separately, with PET SUVR as the dependent variable, the BBM as the primary exposure, and age, sex, race, education, and *APOE* ε4 as covariates. Subsequently, an additional two models were conducted for each BBM to evaluate the impact of adding BMI versus BV as covariates following standardization.

#### 2.6.3 Modification of BBM‐PET SUVR associations by BMI and BV

To assess whether BMI/BV modified associations between BBMs and amyloid PET SUVR, we repeated the analysis after incorporating BMI or BV as interaction terms with BBMs instead of as independent covariates. All else was held constant.

#### 2.6.4 Associations between BBMs and amyloid PET positivity

To assess the predictive capacities of BBMs on amyloid PET positivity, ROC curve analyses were conducted from cross‐sectional data using the baseline visit for each participant. Specifically, a separate logistic regression model was generated for each BBM, with amyloid PET status as the dependent variable, the BBM as the primary exposure, and age, sex, race, education, and *APOE* ε4 as covariates. Subsequently, we evaluated the impact of adding BMI or BV, as covariates or as interaction terms, on area under the curve (AUC) values. DeLong's test was used to assess whether observed differences in AUC were significant after adjusting for BMI/BV, which provides a nonparametric approach for comparing AUCs while accounting for the correlation structure of the data. Since ROC curves were generated from the same set of participants (only covariate addition was tested), the paired version of DeLong's test was used, accounting for within‐subject correlation.

To further evaluate whether ROC performance differed by BMI group, we conducted separate ROC curves in normal‐weight versus overweight/obese participants, enabling separate cutoff thresholds. Since participant groups were non‐overlapping, we used the unpaired version of DeLong's tests to determine whether observed differences in AUC values were significant between groups. The AUC value in each subgroup was also compared with the full cohort, and bootstrapping was used to assess confidence intervals for group differences through resampling (*n* = 5000 iterations). Each of these analyses were repeated after stratifying participants by BMI and BV medians instead of BMI groups.

#### 2.6.5 Influence of BMI and BV when determining BBM cutoff thresholds for PET positivity

To further assess the impact of BMI classification on BBM cutoff thresholds for amyloid PET positivity, we derived optimal ROC‐based cutoffs separately for normal‐weight participants and overweight/obese participants matched on age and amyloid positivity with a caliper of 0.1. After matching, no significant differences were observed between normal‐weight and overweight/obese participants in age, sex, race, education, or *APOE* ε4 status, except for a higher proportion of females in the normal‐weight group for p‐Tau_217_^(Fujirebio)^ (66% [95% CI: 53%–76%] vs. 46% [95% CI: 34%–58%], *p *= 0.045) and p‐Tau_217_^(Quanterix,Alzpath)^ (62% [95% CI: 49%–73%] vs. 40% [95% CI: 28%–52%], *p *= 0.026). Next, we assessed the impact of applying a BBM cutoff threshold derived from normal‐weight participants to the overweight/obese group on amyloid PET positivity misclassification rates. Bootstrapping was used to determine confidence intervals for changes in amyloid PET misclassification rates when applying the foreign cutoff threshold (*n* = 5000 iterations with resampling). These analyses were repeated comparing upper versus lower BMI and BV medians.

#### 2.6.6 Changes in BMI and BV over time, and their associations with changes in BBMs

Changes in BMI and BV (exposures) or BBMs (outcomes) over time were each calculated using the following formula:

(1) $$\begin{array}{c} \frac{X_{visit\;n+1}-\;X_{visit\;n}}{t_{visit\;n+1}-t_{visit\;n}}\# \end{array}$$

where *X* represents BMI, BV or a given BBM, and t refers to the time at a given visit. Difference in time was calculated in years, from visitn to visitn+1.

To assess the impact of changes in BMI or BV on BBM concentrations, we used Formula 1 to calculate the rate of change for each variable. LMMs were generated for each BBM, with the rate of change in BBM concentration as the time‐varying dependent variable and the rate of change in BMI or BV as the primary time‐varying exposure. All models were adjusted for age, sex, race, education, *APOE* ε4 status, and time between visits. Models included random intercepts to account for within‐subject correlation. To further disentangle dilution effects from the impact of early neurodegeneration and systemic comorbidities, analyses were repeated after stratifying by amyloid PET status.

#### 2.6.7 Impact of drastic declines in BMI and BV on PET prediction accuracy

To assess whether substantial declines in BMI or BV affect the bias and accuracy of BBM‐based predictions of amyloid PET SUVR, we compared model performance between participants with significant BMI or BV reductions (> 1 SD below the mean; “decline” group) and those with stable values (within 1 SD; “stable” group), focusing on amyloid‐negative individuals. Participants in the decline and stable groups were matched by age, sex, BMI or BV at first visit, and interval between visits. After matching, no significant group differences were observed in these variables for any analysis. Models were trained on unmatched stable participants then used to predict PET SUVR in matched stable versus decline groups. All training and test models were adjusted for age, sex, race, education, *APOE* ε4 status, and time between visits. Mean error was used to evaluate systematic over‐ or underestimation, while root mean squared error (RMSE) captured overall prediction accuracy and variability. This analysis was performed in both directions: predicting PET SUVR from BBMs and predicting BBMs from PET SUVR, in order to evaluate systematic over‐ or under‐estimation in the decline group.

## 3 RESULTS

### 3.1 Cohort characteristics

**FIGURE 1:**
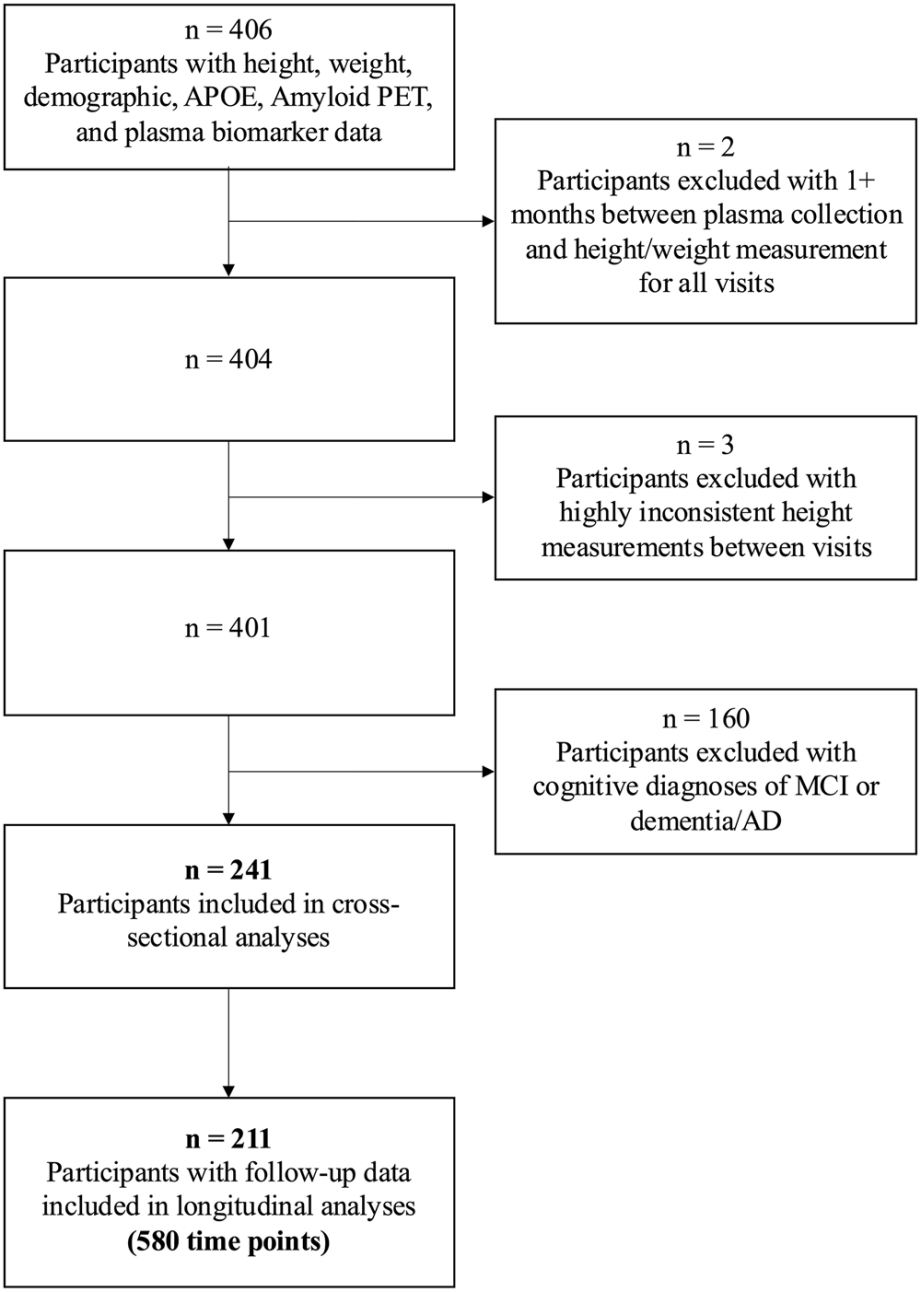
Study exclusion criteria. Participants were included in the analysis based on the availability of height, weight, demographic (sex, race, and education), *APOE*, and BBMs in addition to being classified as CU for the duration of the analysis. APOE, apolipoprotein E; BBM, blood‐based biomarker; CU, cognitively unimpaired.

A total of 241 ADNI participants were included in the study, with 211 having longitudinal data (mean = 2.75 visits per participant, SD = 0.48; average time between visits = 3.05 years, SD = 1.20) (Figure 1). At baseline (defined as the first visit included in this analysis), participants were on average 73.4 years old [SD = 6.8], 53.9% female, 91.7% White individuals, and 29.9% *APOE* ε4 carriers. The average BMI was 27.49 [SD = 4.83], and average BV was 4.65 L [SD = 0.85]. Using CDC standard BMI categorization, only 1 (0.4%) participant was classified as underweight (BMI < 18.5), 71 (29.5%) were classified as normal weight (18.5≤BMI < 25), 113 (46.9%) were classified as overweight (25≤BMI < 30), and 56 (23.2%) were classified as obese (BMI≥30) (Table 1).

Baseline characteristics were compared across normal weight, overweight, and obese participants (Table 1; lean participants were not analyzed since only one participant was in this category). BV was lowest in normal weight participants (4.16 L [SD = 0.77]), intermediate in overweight participants (4.74 L [SD = 0.76]), and highest in obese participants (5.12 L [SD = 0.86]) (*p* < 0.001). Moreover, age differed between groups, with normal weight participants being the oldest (74.5 years [SD = 7.2]), overweight participants being intermediate (73.7 years [SD = 6.8]), and obese participants being the youngest (71.5 years [SD = 6.2]). There were marginally significant differences in the proportion of males between groups (42.3% [95% CI: 31.5%–53.8%], 54.0% [95% CI: 44.8%–62.9%], and 35.7% [95% CI: 24.5%–48.8%], respectively; *p* = 0.059). There were also differences in years of education (16.86 [SD = 2.53], 16.58 [SD = 2.54], 16.34 [SD = 2.49]; *p *= 0.508), and *APOE* ε4 carriers (33.8% [95% CI: 23.9%–45.4%], 31.0% [95% CI: 23.2%–40.0%], 23.2% [95% CI: 14.1%–35.8%]; *p* = 0.413), but these differences were not significant following analysis of variance (ANOVA) (Table 1).

In terms of BBMs, p‐Tau_217_^(C2N)^ was highest in normal weight participants (2.31 [SD = 2.32]), lower in overweight participants (1.86 [SD = 2.05]), and lowest in obese participants (1.37 [SD = 1.29]) (*p* = 0.036). The same pattern was observed for p‐Tau_217_/N‐ptau_217_^(C2N)^ (4.50 [SD = 3.76], 3.29 [SD = 2.56], and 2.61 [SD = 2.00], respectively; *p* = 0.001), NfL^(Roche)^ (4.02 [SD = 1.49], 3.34 [SD = 1.32], and 3.12 [SD = 1.39]; *p* = 0.001), and NfL^(Quanterix)^ (22.24 [SD = 8.61], 17.81 [SD = 8.52], 17.81 [SD = 8.18]; *p *= 0.005). p‐Tau_217_ levels measured by the remaining assay platforms showed similar trends across BMI groups, but reached only marginal significance: Fujirebio (0.20 [SD = 0.26], 0.17 [SD = 0.27], 0.10 [SD = 0.08]; *p* = 0.069), Alzpath (0.40 [SD = 0.27], 0.38 [SD = 0.29], 0.30 [SD = 0.17]; *p *= 0.103), and Janssen (0.06 [SD = 0.04], 0.05 [SD = 0.04], 0.04 [SD = 0.03]; *p *= 0.083). No significant group differences were observed for Aβ_42/40_, p‐Tau_181_, or GFAP across any platforms (Table 1).

Notably, there were no significant differences between normal weight, overweight, or obese participants in amyloid PET SUVR or amyloid PET positivity, suggesting that the above BBM differences by BMI group likely do not reflect brain amyloid burden. Specifically, mean SUVR values were 1.13 [SD = 0.22] in normal weight participants, 1.11 [SD = 0.21] in overweight participants, and 1.06 [SD = 0.15] in obese participants (*p* = 0.175), and the proportion of participants classified as amyloid‐positive in these groups was 35.2% (95% CI: 25.1%–46.8%), 33.6% (95% CI: 25.6%–42.7%), and 21.4% (95% CI: 12.7%–33.8%), respectively (*p* = 0.189) (Table 1).

### 3.2 Continuous associations between BMI/BV and BBMs

In adjusted LMMs encompassing all plasma visits, higher BMI was significantly associated with lower levels of p‐Tau_217_^(C2N)^ (*β* = ‐0.032, *p *= 0.025), p‐Tau_217_/N‐ptau_217(_^C2N)^ (*β *= ‐0.044, *p* < 0.001), p‐Tau_217_^(Fujirebio)^ (*β* = ‐0.034, *p* = 0.034), p‐Tau_217_^(Quanterix, Alzpath)^ (*β* = ‐0.024, *p *= 0.034), GFAP^(Roche)^ (*β* = ‐0.032, *p* ≤ .001), GFAP^(Quanterix)^ (*β* = ‐0.028, *p *= 0.015), NfL^(Roche)^ (*β *= ‐0.050, *p* < 0.001), and NfL^(Quanterix)^ (*β* = ‐0.057, *p *< 0.001). Higher BV associated with lower p‐Tau_217_/N‐ptau_217_^(C2N)^ (*β *= ‐0.054, *p *= 0.006), GFAP^(Roche)^ (*β* = ‐0.048, *p *= 0.002) and GFAP^(Quanterix)^ (*β* = ‐0.043, *p* = 0.016), and NfL^(Roche)^ (*β* = ‐0.074, *p* < 0.001) and NfL^(Quanterix)^ (*β *= ‐0.082, *p *< 0.001), demonstrating larger effect sizes compared to BMI (Table S2).

### 3.3 Associations between BBMs and amyloid PET SUVR

Lower plasma Aβ_42/40_ associated with higher PET SUVR across all assay platforms except C2N, while higher levels of plasma p‐Tau_181_, p‐Tau_217_, p‐Tau_217_/n‐pTau_217_, and GFAP associated with higher PET SUVR (Table S3). After adjusting for BMI as a covariate, these associations remained, except with Aβ_42/40_^(Fujirebio)^ (Table S3). BMI itself was associated with PET SUVR for models in which Aβ_42/40_^(C2N)^, Aβ_42/40_^(Fujirebio)^, and Aβ_42/40_^(Roche)^ were the primary exposures, but not in models with other BBMs. Similarly, associations remained after adjusting for BV as a covariate, except with Aβ_42/40_^(Fujirebio)^. BV itself was not associated with PET SUVR in any BBM models (Table S3).

### 3.4 Modification of BBM‐PET SUVR associations by BMI and BV

Several BMI‐BBM interaction terms associated with amyloid PET SUVR including interactions with Aβ_42/40_^(C2N)^, Aβ_42/40_^(Roche)^, and p‐Tau_217_^(Quanterix, Alzpath)^, while an even greater number of BV‐BBM interaction terms associated with amyloid PET SUVR, including interactions with Aβ_42/40_^(Roche)^, all p‐Tau_181_ measures, all p‐Tau_217_ measures, all GFAP measures, and all NfL measures (Table 2). Moreover, a comparison of standardized β coefficients for BMI and BV showed that BV‐BBM interactions were consistently stronger than BMI‐BBM interactions for all p‐Tau_181_, p‐Tau_217_, GFAP, and NfL biomarkers, indicating that BV may modify the effect of BBMs on amyloid PET more than BMI (Table 2).

**TABLE 2:** Interactions between BMI/BV and BBMs on amyloid PET SUVR outcomes.

**BBM (exposure)**	**Sample size (time points)**	**BMI model**			**BV model**		
		**BBM β ± SE**	**BMI β ± SE**	**BBM** ^*^ **BMI β ± SE**	**BBM β ± SE**	**BV β ± SE**	**BBM** ^*^ **BV β ± SE**
Aβ_42/40_^(C2N)^	206 (559)	−0.38 ± 0.24	**−0.053 ± 0.021** ^*^	**0.43 ± 0.22** ^*^	−0.36 ± 0.25	−0.022 ± 0.025	0.12 ± 0.25
Aβ_42/40_^(Fujirebio)^	206 (559)	**−0.35 ± 0.18** ^*^	−0.025 ± 0.021	0.14 ± 0.22	−0.39 ± 0.19^*^	−0.026 ± 0.019	0.17 ± 0.17
Aβ_42/40_^(Roche)^	204 (551)	**−0.00077 ± 0.00022** ^***^	**−0.078 ± 0.026** ^**^	**0.00052 ± 0.0002** ^*^	**−0.00068 ± 0.00022** ^**^	**−0.078 ± 0.029** ^**^	**0.00055 ± 0.00022** ^*^
Aβ_42/40_^(Quanterix)^	185 (487)	**−0.92 ± 0.39** ^*^	0.022 ± 0.024	−0.5 ± 0.38	−0.66 ± 0.4^†^	−0.02 ± 0.023	0.25 ± 0.34
p‐Tau_181_^(Roche)^	205 (555)	**0.16 ± 0.023** ^***^	−0.01 ± 0.0056^†^	−0.018 ± 0.021	**0.16 ± 0.023** ^***^	−0.0073 ± 0.009	**−0.069 ± 0.019** ^***^
p‐Tau_181_^(Quanterix)^	186 (507)	**0.0014 ± 0.00033** ^***^	0.004 ± 0.0089	−0.00056 ± 0.00032^†^	**0.0015 ± 0.00032** ^***^	**0.034 ± 0.012** ^**^	**−0.0018 ± 0.00034** ^***^
p‐Tau_217_^(C2N)^	206 (559)	**0.12 ± 0.011** ^***^	−0.004 ± 0.0053	−0.015 ± 0.009	**0.12 ± 0.01** ^***^	0.00083 ± 0.0083	**−0.031 ± 0.0093** ^***^
p‐Tau_217_/N‐ptau_217_^(C2N)^	206 (558)	**0.12 ± 0.011** ^***^	0.00083 ± 0.0065	−0.018 ± 0.0093^†^	**0.12 ± 0.011** ^***^	0.0091 ± 0.0091	**−0.034 ± 0.0097** ^***^
p‐Tau_217_^(Fujirebio)^	206 (559)	**0.11 ± 0.011** ^***^	**−0.021 ± 0.01** ^*^	−0.015 ± 0.0095	**0.12 ± 0.011** ^***^	**−0.052 ± 0.013** ^***^	**−0.049 ± 0.01** ^***^
p‐Tau_217_^(Quanterix,Alzpath)^	206 (540)	**0.21 ± 0.016** ^***^	**−0.018 ± 0.0079** ^*^	**−0.026 ± 0.013** ^*^	**0.2 ± 0.016** ^***^	**−0.026 ± 0.01** ^*^	**−0.046 ± 0.013** ^***^
p‐Tau_217_^(Quanterix,Janssen)^	206 (541)	**0.15 ± 0.016** ^***^	**−0.037 ± 0.019** ^*^	−0.023 ± 0.014^†^	**0.15 ± 0.017** ^***^	**−0.069 ± 0.02** ^***^	**−0.049 ± 0.014** ^***^
GFAP^(Roche)^	205 (554)	**0.098 ± 0.022** ^***^	−0.032 ± 0.018^†^	−0.024 ± 0.017	**0.1 ± 0.022** ^***^	**−0.055 ± 0.019** ^**^	**−0.049 ± 0.016** ^**^
GFAP^(Quanterix)^	185 (487)	**0.052 ± 0.021** ^*^	0.048 ± 0.036	−0.026 ± 0.017	**0.052 ± 0.02** ^*^	**0.094 ± 0.034** ^**^	**−0.046 ± 0.016** ^**^
NfL^(Roche)^	205 (554)	0.023 ± 0.023	0.0043 ± 0.011	−0.028 ± 0.018	0.022 ± 0.023	0.016 ± 0.014	**−0.044 ± 0.019** ^*^
NfL^(Quanterix)^	185 (487)	0.00087 ± 0.022	0.025 ± 0.021	−0.027 ± 0.016^†^	0.0014 ± 0.022	**0.049 ± 0.024** ^*^	**−0.043 ± 0.018** ^*^

*Note*: All models were linear mixed effects models incorporating random effects to account for within‐participant correlation across visits. Models were adjusted for age, sex, race, education, and *APOE* ε4. The BMI interaction model included an additional interaction term between BMI and the respective BBM exposure, while the BV interaction model included BV as an interaction term with the respective BBM exposure. All non‐amyloid plasma biomarkers were log10 transformed to improve skewness and kurtosis. BMI and BV were standardized to compare relative strengths of association. Significance levels are denoted as: ^†^ *p* ≤ 0.100; ^*^ *p* ≤ 0.050; ^**^ *p* ≤ 0.010; ^***^ *p* ≤ 0.001.

Abbreviations: Aβ, amyloid β; APOE, apolipoprotein E; BBM, blood‐based biomarker; BMI, body mass index; BV, blood volume; GFAP, glial fibrillary acidic protein; NfL, neurofilament light; N‐ptau, non‐phosphorylated tau; p‐Tau, phosphorylated tau; PET, positron emission tomography; SE, standard error; SUVR, standardized uptake value ratio

**FIGURE 2:**
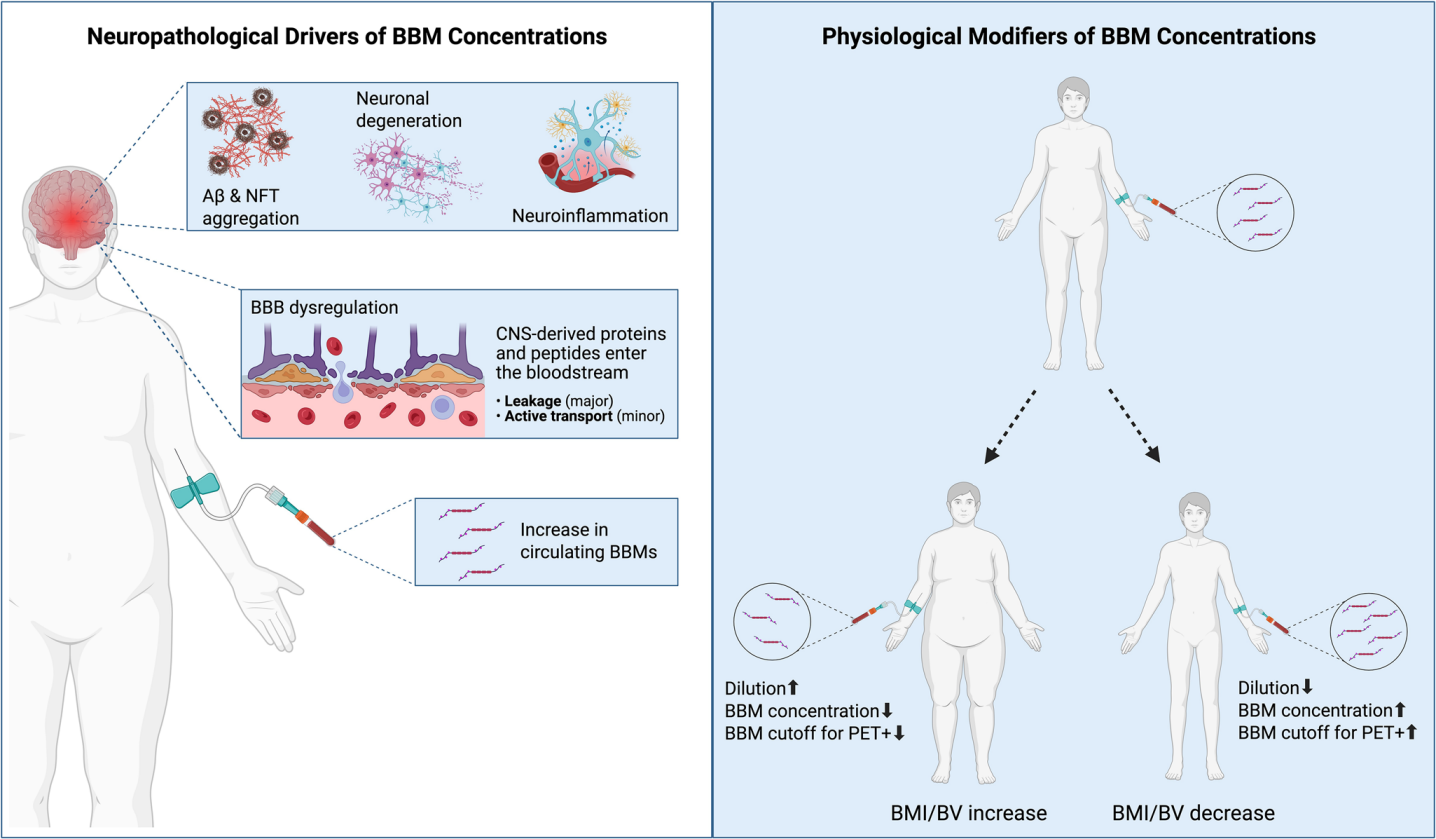
Neuropathological and physiological contributions to BBM concentrations. The left panel illustrates how AD‐related pathology, including amyloid and tau aggregation, neurodegeneration, and neuroinflammation, leads to increased release of CNS‐derived biomarkers into the blood, facilitated by BBB dysregulation. The right panel shows how physiological factors such as BMI and BV can influence BBM concentrations through dilution effects, potentially lowering circulating levels in the absence of pathological change and impacting interpretation of PET‐based BBM thresholds. Aβ, amyloid‐β; BBB, blood–brain barrier; BBM, blood‐biomarker; BMI, body mass index; BV, blood volume; CNS, central nervous system; NFT, neurofibrillary tangles; PET, positron emission tomography. Created in BioRender. Jacobs, T. (2025) https://BioRender.com/yhiqean.

Importantly, these interaction terms were consistently in the opposite direction of the corresponding BBM‐PET associations. For example, interactions between BMI/BV and Aβ_42/40_ with PET SUVR were positive, while interactions between BMI/BV and p‐Tau_181_, p‐Tau_217_, GFAP, and NfL with PET SUVR were negative. This pattern indicated that in individuals with higher BMI or BV, the cross‐sectional associations between BBMs and PET SUVR were attenuated, with slopes becoming less steep, that is, the regression line between BBMs and PET SUVR flattened at higher BMI or BV, consistent with dilution effects (Table 2, Figure 2).

### 3.5 Associations between BBMs and amyloid PET positivity

Shifting from SUVR to amyloid PET positivity, adding BMI or BV as independent covariates or interaction terms provided little to no improvement in AUC values compared to models with BBMs alone, and none of these changes in AUC were significant (Table S4).

**FIGURE 3:**
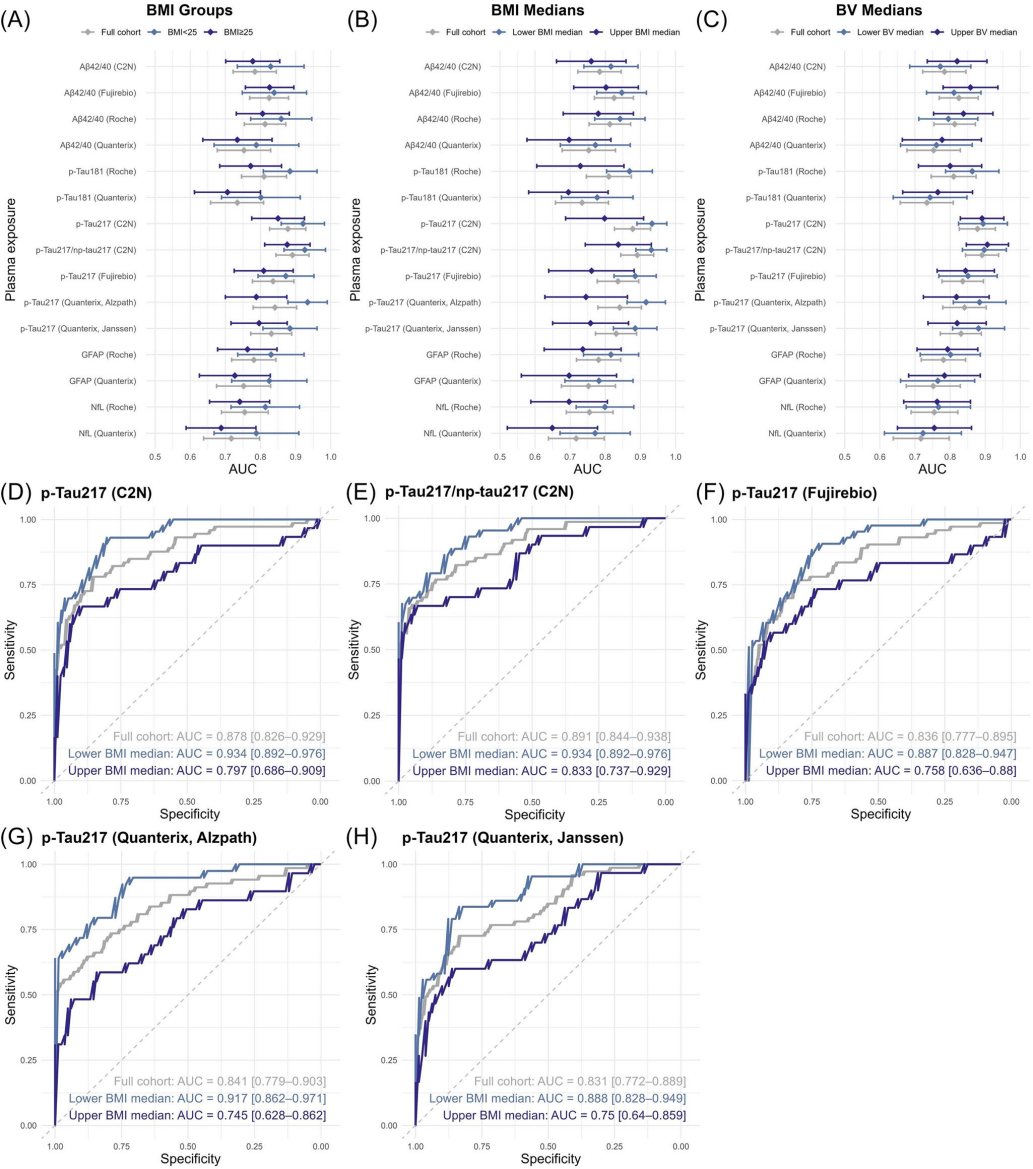
ROC curve results across plasma biomarkers on predicting amyloid PET positivity, a comparison of BMI and BV groups. ROC curves were analyzed for all BBMs on predicting amyloid PET positivity. AUC results were compared (A) between normal weight versus overweight/obese participants, (B) between the lower and upper BMI medians, and (C) between the lower and upper BV medians. In panels (A)–(C), diamonds denote AUC, and error bars denote the 95% CI. The most drastic differences were seen in all p‐Tau_217_ markers, especially when comparing BMI medians (D)–(H). All models were adjusted for age, sex, race, education, and *APOE* ε4. APOE, apolipoprotein E; AUC, area under the curve; BBM, blood‐based biomarker; BMI, body mass index; BV, blood volume; CI, confidence interval; PET, positron emission tomography; ROC, receiver operating characteristics.

However, when conducting separate ROC analyses between BMI subgroups, normal‐weight participants consistently had higher AUC values across all BBMs compared to overweight and obese participants (Figure 3A, Table S5). These group differences in AUC were significant for p‐Tau_217_^(Quanterix, Alzpath)^ (0.934 [95% CI: 0.878–0.990] vs 0.788 [95% CI: 0.700–0.875], respectively; *p* = 0.006) and marginally significant for p‐Tau_181_^(Roche)^ (0.884 [95% CI: 0.807–0.960] vs 0.772 [95% CI: 0.684–0.860]; *p *= 0.060) (Table S5). Similarly, participants in the lower BMI median consistently had higher AUC values than those in the upper BMI median, and these differences were significant for p‐Tau_181_^(Roche)^ (0.872 [95% CI: 0.808–0.936] vs 0.727 [95% CI: 0.602–0.851], respectively; *p *= 0.043), p‐Tau_217_^(C2N)^ (0.934 [95% CI: 0.892–0.976] vs 0.797 [95% CI: 0.686–0.909]; *p *= 0.026), p‐Tau_217_^(Quanterix, Alzpath)^ (0.917 [95% CI: 0.862–0.971] vs 0.745 [95% CI: 0.628–0.862]; *p *= 0.010), and p‐Tau_217_^(Quanterix, Janssen)^ (0.888 [95% CI: 0.828–0.949] vs 0.750 [95% CI: 0.640–0.859]; *p *= 0.031), and marginally significant for p‐Tau_181_^(Quanterix)^ (0.797 [95% CI: 0.701–0.893] vs 0.666 [95% CI: 0.546–0.786]; *p *= 0.099), p‐Tau_217_/N‐ptau_217_^(C2N)^ (0.934 [95% CI: 0.892–0.976] vs 0.833 [95% CI: 0.737–0.929]; *p *= 0.062), p‐Tau_217_^(Fujirebio)^ (0.887 [95% CI: 0.828–0.947] vs 0.758 [95% CI: 0.636–0.880]; *p *= 0.063), and NfL^(Roche)^ (0.806 [95% CI: 0.727–0.885] vs 0.690 [95% CI: 0.578–0.801]; p = 0.094) (Figure 3B and 3D–H, Table S5). Results were less consistent when stratified by BV, with neither median showing consistently greater or significantly different AUC values (Figure 3C, Supplementary Table 5).

Lastly, AUC values from each BMI/BV subgroup were compared with the full cohort. For every BBM, BMI and BV stratified ROC analysis consistently demonstrated greater AUC values than the full cohort in at least one subgroup or median. However, differences were generally not significant within a 95% CI (Figure 3, Table S6).

### 3.6 Influence of BMI and BV when determining BBM cutoff thresholds for PET positivity

**FIGURE 4:**
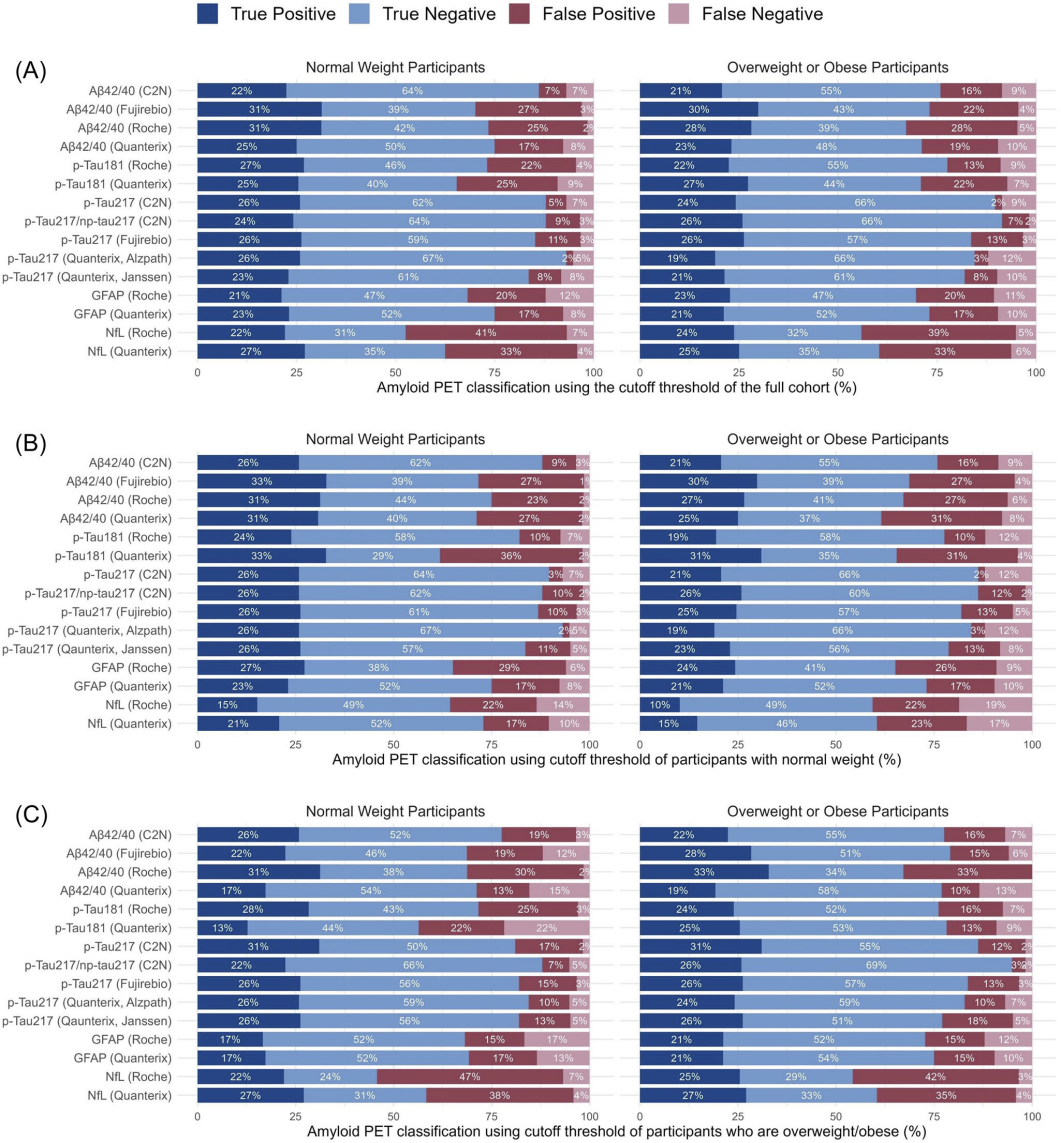
PET classification accuracies by BBM cutoff thresholds and BMI group. Amyloid PET classification was assessed using BBM cutoff thresholds derived from (A) the full cohort, (B) normal‐weight participants, and (C) overweight/obese participants. To evaluate the impact of applying thresholds from one BMI group to another, each cutoff was used to predict amyloid PET status in both normal‐weight (left) and overweight/obese participants (right). Normal‐weight participants were independently matched to overweight/obese participants based on age and amyloid positivity status. BBM, blood‐based biomarker; PET, positron emission tomography.

Overweight/obese participants had lower PET positivity cutoff thresholds compared to matched normal‐weight participants for all p‐Tau_217_ and NfL measures and higher thresholds for all GFAP measures, while Aβ_42/40_ and p‐Tau_181_ thresholds showed no consistent pattern (Table S7). Applying thresholds derived from normal‐weight participants to matched overweight/obese participants increased overall misclassification rates for all BBMs and false negative rates for all p‐Tau_217_ and NfL measures (Figure 4). Increases were significant in false negative rates for p‐Tau_217_^(C2N)^ (mean difference: 28%, 95% CI: 5.0%–52.6%), false positive rates for Aβ_42/40_^(Fujirebio)^ (20.0%, 95% CI: 2.4%–35.7%) and p‐Tau_217_/N‐pTau_217_^(C2N)^ (13.3%, 95% CI: 2.8%–25.6%), and overall misclassification rates for Aβ_42/40_^(Fujirebio)^ (11.7%, 95% CI: 2.9%–22.4%), Aβ_42/40_^(Quanterix)^ (14.9%, 95% CI: 1.9%–30.8%), and p‐Tau_217_/N‐pTau_217_^(C2N)^ (9.5%, 95% CI: 1.7%–19.0%) (Table S8). Conversely, applying thresholds derived from overweight/obese participants to matched normal‐weight participants increased the overall misclassification rates for all BBMs except Aβ_42/40_^(Quanterix)^ and p‐Tau_217_/np‐Tau_217_^(C2N)^, and increased the false positive rates for all p‐Tau_217_ and NfL values (Figure 4). Increases were significant in false negative rates for Aβ_42/40_^(Quanterix)^ (mean difference: 37.3%, 95% CI: 7.1‐65.2%), false positive rates for Aβ_42/40_^(Roche)^ (13.5%, 95% CI: 2.4‐29.2%), and overall misclassification rates for Aβ_42/40_^(Roche)^ (8.6%, 95% CI: 1.6‐17.2%) and p‐Tau_217_^(Quanterix, Alzpath)^ (8.4%, 95% CI: 1.7‐17.2%) (Table S8). Lastly, applying thresholds derived from the full‐cohort instead of group‐specific thresholds also increased misclassification rates (Figure 4, Table S8), but differences were not significant.

Similarly, increases in false negative rates, false positive rates, and overall misclassification rates were observed when thresholds were switched between matched participants in the lower and upper BMI medians (Table S9), as well as between those in the upper and lower BV medians (Table S10). Applying the full cohort cutoff threshold instead of median‐specific thresholds increased misclassification rates (Tables S9, S10), but differences were generally not significant.

### 3.7 Changes in BMI and BV over time, and their associations with changes in BBMs

Greater increases in BMI over time significantly associated with greater decreases in p‐Tau_217_^(Quanterix, Alzpath)^, p‐Tau_217_^(Quanterix, Janssen)^, GFAP^(Roche)^, and all NfL measures (Table 3). Other than for p‐Tau_217_^(Quanterix, Janssen)^, each of these associations persisted after adjusting for changes in amyloid PET SUVR, suggesting they were independent of changes in brain amyloid burden (Table 3). Similarly, greater increases in BV over time were significantly associated with greater decreases in p‐Tau_217_^(Quanterix, Alzpath)^, p‐Tau_217_^(Quanterix, Janssen)^, all GFAP measures, and all NfL measures (Table 3), and each of these associations remained significant after adjusting for changes in amyloid PET SUVR. A comparison of standardized β coefficients indicated that relative to BMI changes, BV changes consistently exhibited stronger associations with BBM changes before and after adjusting for PET (Table 3).

**TABLE 3:** Associations between changes in BMI or BV with changes in BBMs over time.

**BBM (outcome)**	**Sample size (time points)**	Without PET adjustment		With PET adjustment	
		BMI β ± SE	BV β ± SE	BMI β ± SE	BV β ± SE
Aβ_42/40_^(C2N)^	205 (349)	−0.0002 ± 0.00024	−0.00025 ± 0.00024	−0.00023 ± 0.00024	−0.00026 ± 0.00024
Aβ_42/40_^(Fujirebio)^	205 (349)	0.00015 ± 0.00029	0.00011 ± 0.00029	0.00016 ± 0.00029	0.00012 ± 0.00029
Aβ_42/40_^(Roche)^	202 (343)	−0.36 ± 0.24	−0.41 ± 0.24†	−0.35 ± 0.24	−0.41 ± 0.24†
Aβ_42/40_^(Quanterix)^	173 (296)	−0.00021 ± 0.00017	−0.00019 ± 0.00017	−0.00021 ± 0.00017	−0.00018 ± 0.00017
p‐Tau_181_^(Roche)^	203 (346)	−0.0024 ± 0.002	−0.0028 ± 0.002	−0.0023 ± 0.0021	−0.0027 ± 0.002
p‐Tau_181_^(Quanterix)^	179 (315)	−0.018 ± 0.14	−0.02 ± 0.14	0.016 ± 0.14	−0.00037 ± 0.14
p‐Tau_217_^(C2N)^	205 (349)	−0.0032 ± 0.0042	−0.0071 ± 0.0042†	−0.0028 ± 0.0043	−0.0069 ± 0.0042
p‐Tau_217_/N‐ptau_217_^(C2N)^	205 (348)	0.00028 ± 0.0043	−0.002 ± 0.0043	0.00095 ± 0.0043	−0.0017 ± 0.0043
p‐Ttau_217_^(Fujirebio)^	205 (349)	−0.0029 ± 0.0037	−0.0039 ± 0.0037	−0.0025 ± 0.0037	−0.0038 ± 0.0037
p‐Tau_217_^(Quanterix,Alzpath)^	196 (327)	**−0.0051 ± 0.0022** ^*^	**−0.006 ± 0.0022** ^**^	**−0.0046 ± 0.0022** ^*^	**−0.0057 ± 0.0022** ^**^
p‐Tau_217_^(Quanterix,Janssen)^	200 (328)	**−0.0057 ± 0.0028** ^*^	**−0.0068 ± 0.0028** ^*^	−0.0053 ± 0.0029†	**−0.0066 ± 0.0029** ^*^
GFAP^(Roche)^	203 (345)	**−0.0046 ± 0.0022** ^*^	**−0.0047 ± 0.0023** ^*^	**−0.0047 ± 0.0023** ^*^	**−0.0048 ± 0.0023** ^*^
GFAP^(Quanterix)^	173 (296)	−0.0041 ± 0.0029	**−0.0058 ± 0.0029** ^*^	−0.0047 ± 0.0029	**−0.0059 ± 0.0029** ^*^
NfL^(Roche)^	203 (346)	**−0.0071 ± 0.0024** ^**^	**−0.0075 ± 0.0024** ^**^	**−0.0073 ± 0.0024** ^**^	**−0.0076 ± 0.0024** ^**^
NfL^(Quanterix)^	173 (296)	**−0.0099 ± 0.0029** ^***^	**−0.011 ± 0.0029** ^***^	**−0.01 ± 0.0029** ^***^	**−0.011 ± 0.0029** ^***^

*Note*: Each outcome represents change over time for the given BBM, based on Formula 1. Similarly, BMI represents change in BMI, and BV represents change in BV, with each determined using Formula 1. Change in BMI and change in BV were z‐score standardized for comparison. Change in BMI was the primary exposure in the BMI models, and change in BV was the primary exposure in the BV models. All models were adjusted for age, sex, race, education, and *APOE* ε4. Models indicated as PET adjusted had changes in amyloid PET SUVR added as an additional covariate. All non‐Aβ_42/40_ plasma biomarkers were log10 transformed to improve skewness and kurtosis. Significance levels are denoted as: ^†^ *p* ≤ 0.100; ^*^ *p* ≤ 0.050; ^**^ *p* ≤ 0.010; ^***^ *p* ≤ 0.001.

Abbreviations: Aβ, amyloid β; BBM, blood‐based biomarker; BMI, body mass index; BV, blood volume; GFAP, glial fibrillary acidic protein; NfL, neurofilament light; N‐ptau, non‐phosphorylated tau; PET, positron emission tomography; p‐Tau, phosphorylated tau; SE, standard error.

Among amyloid‐negative participants (*n* = 166), both the number and strength of significant associations were greater than in the full cohort, with larger β coefficients observed for both BMI‐ and BV‐related changes. Specifically, greater increases in BMI over time were significantly associated with greater decreases in Aβ_42/40_^(Roche)^, Aβ_42/40_^(Quanterix)^, p‐Tau_217_^(Quanterix, Alzpath)^, GFAP^(Roche)^, and all NfL measures (Table S11). These associations persisted after adjusting for changes in amyloid PET SUVR, and an additional significant association was identified with p‐Tau_217_^(Quanterix, Janssen)^. Similarly, greater increases in BV over time associated with greater decreases in all Aβ_42/40_ measures except Aβ_42/40_^(Fujirebio)^, all p‐Tau_217_ measures except p‐Tau_217_^(Fujirebio)^, all GFAP measures, and all NfL measures (Table S11). After adjusting for changes in amyloid PET, all associations remained significant except for Aβ_42/40_^(C2N)^, which became marginally significant, while p‐Tau_217_^(Fujirebio)^ reached significance. Comparisons of standardized β coefficients again showed that compared to BMI, changes in BV had stronger associations with changes in BBMs (Table S11).

In contrast, among amyloid‐positive participants (*n* = 75), no significant associations were observed between changes in BMI or BV and changes in any BBM, except for Aβ_42/40_^(Roche)^, which demonstrated an inverse association with changes in BMI before and after adjusting for changes in amyloid PET (Table S11).

### 3.8 Impact of drastic declines in BMI and BV on PET prediction accuracy

Participants in the BMI decline group exhibited no significant difference in BMI at previous visit, but a significantly lower average BMI at follow‐up compared to the stable group in all BBM analyses except Aβ_42/40_^(Roche)^ and p‐Tau_181_^(Quanterix)^ (Table 4, Table S12).

**TABLE 4:** Comparison of prediction model performances in participants with drastic BMI declines versus stable controls.

**BBM**	**N, each group**	**BMI at follow‐up**			**Mean error of predicted PET SUVR**			**Mean error of predicted BBM concentration**		
		**Stable**	**Decline**	***p‐*value**	**Stable**	**Decline**	**Decline—stable [95% CI]**	**Stable**	**Decline**	**Decline—stable [95% CI]**
Aβ_42/40_^(C2N)^	24	28.62	25.78	**0.001** ^***^	0.0059	0.0103	0.0044 [‐0.0183, 0.0268]	0.0007	0.0049	0.0042 [‐0.0010, 0.0093]
Aβ_42/40_^(Fujirebio)^	24	28.62	25.78	**0.001** ^***^	0.0065	0.0078	0.0013 [‐0.0218, 0.0233]	0.004	0.0018	−0.0022 [‐0.0088, 0.0036]
Aβ_42/40_^(Roche)^	23	28.63	26.68	0.194	0.0086	0.0137	0.0051 [‐0.0152, 0.0254]	−0.2304	3.1106	3.3410 [‐5.2970, 12.4813]
Aβ_42/40_^(Quanterix)^	22	28.55	25.99	**0.012** ^*^	0.0103	0.0172	0.0069 [‐0.0155, 0.0313]	0.0011	0.0017	0.0006 [‐0.0042, 0.0055]
p‐Tau_181_^(Roche)^	24	28.31	25.78	**0.001** ^***^	0.0047	0.0142	0.0095 [‐0.0150, 0.0343]	0.0138	−0.0554	−0.0692 [‐0.1460, 0.0147]
p‐Tau_181_^(Quanterix)^	23	27.26	25.95	0.157	−0.0015	0.0119	0.0134 [‐0.0080, 0.0354]	1.6003	−1.4338	−3.0341 [‐9.3408, 3.1444]
p‐Tau_217_^(C2N)^	24	28.62	25.78	**0.001** ^***^	−0.0012	0.0117	0.0129 [‐0.0094, 0.0346]	0.099	−0.054	−0.1530 [‐0.2603, ‐0.0438]*
p‐Tau_217_/N‐ptau_217_^(C2N)^	24	28.62	25.78	**0.001** ^***^	−0.0015	0.0089	0.0103 [‐0.0129, 0.0338]	0.1069	0.0011	−0.1058 [‐0.2087, ‐0.0047]*
p‐Tau_217_^(Fujirebio)^	24	28.62	25.78	**0.001** ^***^	0.0025	0.0131	0.0106 [‐0.0126, 0.0348]	0.0478	−0.0713	−0.1191 [‐0.2587, 0.0272]
p‐Tau_217_^(Quanterix,Alzpath)^	22	28.52	26.05	**0.018** ^*^	0.0111	0.0185	0.0073 [‐0.0203, 0.0353]	−0.0165	−0.0652	−0.0487 [‐0.1728, 0.0679]
p‐Tau_217_^(Quanterix,Janssen)^	24	29.55	26.43	**0.040** ^*^	0.0153	0.0117	−0.0037 [‐0.0277, 0.0210]	−0.0318	−0.061	−0.0292 [‐0.1156, 0.0633]
GFAP^(Roche)^	24	29.64	26.78	**0.041** ^*^	0.0125	0.0149	0.0025 [‐0.0226, 0.0278]	0.1009	0.0002	−0.1007 [‐0.1766, ‐0.0261]*
GFAP^(Quanterix)^	22	28.55	25.99	**0.012** ^*^	0.0055	0.0128	0.0073 [‐0.0169, 0.0326]	0.0334	0.0026	−0.0309 [‐0.1327, 0.0711]
NfL^(Roche)^	24	28.31	25.78	**0.001** ^***^	0.0029	0.0036	0.0007 [‐0.0240, 0.0248]	0.0451	−0.1107	−0.1558 [‐0.2420, ‐0.0684]*
NfL^(Quanterix)^	22	28.55	25.99	**0.012** ^*^	0.0094	0.0124	0.0029 [‐0.0199, 0.0266]	0.0524	−0.079	−0.1314 [‐0.2377, ‐0.0232]*

*Note*: Participants in the stable group had changes in BMI within 1 standard deviation of the mean, while participants in the decline group had decreases in BMI greater than 1 standard deviation from the mean. The stable group was matched to the decline group by age and sex. No statistically significant differences were observed between groups for age, sex, race, education, or *APOE* ε4. First, BBM concentrations were used to predict amyloid PET SUVR in each group. Subsequently, the analysis was reversed, and amyloid PET SUVR values were used to predict BBM concentrations in each group. Mean prediction errors were compared between groups to assess systematic bias. All prediction models were trained on separate, unmatched stable participants. All models were adjusted for age, sex, and *APOE* ε4. All non‐Aβ_42/40_ plasma biomarkers were log10 transformed to improve skewness and kurtosis. All participants were amyloid‐negative. Confidence intervals for group differences were determined via bootstrapping with 5,000 iterations. Significance is denoted by: ^†^90% CI; ^*^ *p* ≤ 0.050 (or 95% CI excludes 0); ^**^ *p* ≤ 0.010; ^***^ *p* ≤ 0.001.

Abbreviations: Aβ, amyloid β; APOE, apolipoprotein E; BBM, blood‐based biomarker; BMI, body mass index; CI, confidence interval; GFAP, glial fibrillary acidic protein; NfL, neurofilament light; N‐ptau, non‐phosphorylated tau; p‐Tau, phosphorylated tau; PET, positron emission tomography; SUVR, standard uptake value ratio.

When predicting amyloid PET SUVR based on BBMs, mean prediction errors were consistently positive within the decline group for all BBMs, and error values were higher compared to the stable group for all BBMs except p‐Tau_217_^(Quanterix, Janssen)^, indicating systematic overestimation in the decline group (Table 4). Likewise, RMSE values were higher in the decline group for all Aβ_42/40_ measures except Aβ_42/40_^(Quanterix)^, all p‐Tau_181_ and p‐Tau_217_ measures except p‐Tau_217_/N‐ptau_217_^(C2N)^, and lower for all GFAP and NfL measures (Table S12). Despite these consistent trends, differences in mean errors and RMSE were not significant within 95% CIs, potentially due to sample size (*n* = 44–48, depending on the analysis). After reversing the analysis to predict BBM concentrations based on amyloid PET SUVR, mean prediction errors within the decline group were consistently positive for all Aβ_42/40_ and GFAP measures and consistently negative for all p‐Tau and NfL measures except p‐Tau_217_/N‐ptau_217_^(C2N)^ (Table 4). Moreover, mean prediction errors for all non‐Aβ_42/40_ BBMs were lower in the decline group compared to the stable group, indicating systematic underestimation in participants with drastic BMI decline. These underestimations compared to the stable group were significant within 95% CIs for p‐Tau_217_^(C2N)^, p‐Tau_217_/N‐ptau_217_^(C2N)^, GFAP^(Roche)^, and all NfL markers (Table 4). Similarly, RMSE values were higher in the decline group for all BBMs except Aβ_42/40_^(Quanterix)^, p‐Tau_217_/N‐pTau_217_^(C2N)^, and GFAP^(Roche)^, but not within 95% CIs (Table S12).

Participants in the BV decline group exhibited no significant difference in BV at previous visit. Importantly, while BV at follow‐up was lower in the decline group, the difference was not statistically significant compared to the stable group, highlighting the imperfect nature of this comparison and potentially attenuating observed effects (Table S13).

Unlike the BMI analysis, mean prediction errors for amyloid PET SUVR based on BBMs were consistently positive in both the stable and decline group, and differences in mean errors and RMSEs for predicted amyloid PET SUVR were less consistent between the stable and decline groups (Table S13). Still, after reversing the analysis to predict BBM concentrations based on amyloid PET SUVR, mean prediction errors for BBMs were consistently negative for all p‐Tau_181_, p‐Tau_217_, and NfL measures in the decline group (Table S13), and mean prediction errors were higher compared to the stable group for all p‐Tau_181_, p‐Tau_217_, and NfL measures, except p‐Tau_217_/N‐ptau_217_^(C2N)^, indicating systematic underestimation in participants with drastic BV decline (Table S13). These underestimations compared to the stable group were significant within a 95% CI for NfL^(Roche)^, and within a 90% CI for p‐Tau_217_^(Quanterix, Janssen)^. Finally, RMSE values were higher in the decline group for all p‐Tau_181_, p‐tau_217_, and NfL measures (Table S13), indicating that BV declines may reduce the accuracy of prediction for these BBMs. However, this higher variability was only significant within a 95% CI for p‐Tau_217_^(Fujirebio)^ and within a 90% CI for NfL^(Roche)^.

## 4 DISCUSSION

In this study, we found that BMI and BV associate dynamically with BBMs, including Aβ_42/40_, p‐Tau_181_, p‐Tau_217_, GFAP, and NfL, providing evidence that these associations are, in part, due to dilution effects.^24^, ^26^, ^27^ Our results highlight the importance of considering BMI or BV when utilizing BBMs for amyloid PET prediction and ATN/IVS classification, especially p‐Tau_217_ or NfL. By contrast, the weaker effects observed for Aβ_42/40_ may partly reflect the higher intrinsic measurement variability of this ratio, which reduces effect sizes and may obscure associations with BMI or BV. Moreover, our results showed that changes in BMI or BV were associated with changes in BBM concentrations even after adjusting for changes in amyloid PET, suggesting that dilution effects persist irrespective of changes in brain amyloid burden in early disease stages. These changes are less apparent in amyloid‐PET positive individuals, likely due to the stronger influence of disease‐driven biomarker elevations, AD‐related weight‐loss, and/or comorbidities overriding dilution effects. Notably, our findings indicate that, compared to BMI, BV generally demonstrated a more robust and persistent influence on BBMs and BBM‐PET associations, suggesting that BV indices may be a more relevant factor in BBM interpretation and adjustment. Properly accounting for BMI and BV can minimize misclassification of amyloid PET status, improve disease tracking, and enable earlier intervention. In this context, it is worth noting that dilution effects may differentially impact BBMs: because disease progression lowers Aβ_42/40_ but raises p‐Tau, dilution could exaggerate downward shifts in Aβ_42/40_ and thereby increase false positives, while dampening elevations in p‐tau and increasing false negatives. Thus, directionality should be considered when evaluating dilution effects and their diagnostic impact.

The inverse associations we observed between BMI/BV and BBMs at baseline, even after adjusting for amyloid PET, align with previous reports.^12^, ^24^, ^25^, ^26^, ^27^, ^28^, ^29^, ^30^, ^31^ For example, Manouchehrinia et al. found BMI and BV inversely associated with NfL concentrations in plasma, but not CSF,^27^ suggesting dilution effects primarily impact BBMs. Similarly, Pichet Binette et al. reported lower plasma NfL concentrations with higher BMI even after adjusting for amyloid PET,^26^ and Yun et al. found that among amyloid PET‐positive individuals, those with higher p‐Tau_217_ burden had lower BMIs.^30^ Notably, our findings align with a recent analysis of the ADNI cohort which reported inverse associations between BMI and BBMs at baseline without excluding cognitively impaired participants.^31^ This indicates these associations may extend across the broader AD continuum, a possibility that future studies could examine through disaggregated analyses. The previous study also found an inverse association between BMI and amyloid PET, which we did not observe, likely because our analyses excluded cognitively impaired participants to reduce confounding. Moreover, the previous study reported that participants with baseline obesity showed greater longitudinal increases in BBMs, and our findings suggest these increases may have been driven, at least in part, by weight loss in participants who were obese at baseline, highlighting the importance of accounting for BMI changes over time.

Consistent with previous reports,^12^, ^26^ we found that adding BMI as an independent covariate provided little to no impact on BBM‐PET SUVR associations, and the same was true for BV. However, our interaction analyses revealed that BMI and BV significantly modulate the relationship between BBMs and amyloid PET SUVR. This distinction suggests that BMI and BV do not directly influence amyloid pathology but rather alter the detectability of circulating BBMs. Furthermore, interactions were stronger and more consistent for BV than for BMI, with more BBMs reaching significance and larger standardized β coefficients, providing additional evidence for a dilution effect rather than a general metabolic or body composition confound. Specifically, BV‐BBM interactions were significant across p‐Tau_181_, p‐Tau_217_, GFAP, and NfL assays, with their negative direction indicating that higher BV attenuates the positive associations between these BBMs and amyloid PET. Together, these results support the presence of dilution effects, wherein individuals with greater BV exhibit lower BBM concentrations for the same underlying pathology.

Shifting to clinical implications within the ATN/IVS framework, our findings align with prior research demonstrating that including BMI or BV as static covariates yields minimal improvement in predicting PET classification.^12^, ^26^ Similarly, incorporating BMI or BV measures as interaction terms with BBMs did not significantly enhance classification prediction. Although interaction effects were significant when predicting continuous SUVR outcomes, their lack of impact on PET classification likely reflects the use of a single global cutoff applied to all participants in full‐cohort ROC models, as opposed to BMI‐ or BV‐specific BBM thresholds. Therefore, BMI‐ and BV‐stratified analyses were performed.

Accordingly, defining BBM cutoff thresholds for PET positivity within distinct BMI groups improved classification prediction. ROC analyses showed that normal‐weight participants consistently exhibited higher AUC values in predicting amyloid PET positivity compared to overweight/obese participants. Additionally, optimal p‐Tau_217_ and NfL cutoff thresholds were consistently higher in normal‐weight compared to overweight/obese participants, suggesting dilution effects. Applying group‐specific cutoffs reduced misclassification rates for nearly all BBMs after matching participants by age, sex, and amyloid PET status. These findings support the use of BMI‐stratified thresholds in BBM‐based AD diagnostics. Although classification improvements were only significant for certain BBMs, many others had 95% confidence intervals that included zero at the boundary, suggesting marginal significance. This may have been due to the limited number of PET amyloid‐positive observations and few participants classified as obese after matching, resulting in subtle threshold differences, minimal discordance, and reduced observable benefit of applying BMI‐stratified BBM thresholds on PET classification. Therefore, we encourage future studies with larger, obesity‐enriched cohorts to further evaluate the clinical relevance of physiologically informed thresholds. Such studies could better assess the impact of group‐specific cutoff adjustments on PET classification and determine whether BMI/BV‐informed thresholds are particularly beneficial for individuals with BBM values near positivity cutoffs, where small dilution‐related shifts could alter classification. For BBMs with high overall performance, BMI adjustment may be less critical when values lie well above or below the threshold.

In addition to static measures, our results demonstrate that changes in BMI and BV over time, but especially BV, directly influence changes in BBM concentrations. These findings align with a recent study by Rebelos et al., which demonstrated that individuals with morbid obesity had lower plasma NfL and GFAP concentrations compared to controls, but concentrations increased to match control values following weight‐loss surgery.^29^ Moreover, the persistence of our findings after adjusting for changes in PET SUVR suggests they are independent of pathological burden. We observed that these effects were stronger in amyloid‐negative participants and nearly absent in amyloid‐positive participants, suggesting dilution effects are more relevant in earlier disease stages. As the disease progresses, BBM elevations driven by AD‐related neurodegenerative processes, co‐pathologies, and associated weight loss may overshadow these effects. Similarly, our findings suggest that significant declines in BMI and BV introduce systematic misclassification and variability within BBM‐PET predictions. Although our analysis on drastic BV decline was limited by the inability to detect a meaningful decline group with significantly lower BV at follow‐up compared to matched stable controls, prediction errors of BBMs based on amyloid PET in the BV decline group were still directionally consistent with those observed in the BMI decline group, especially for p‐Tau_181_, p‐Tau_217_, and NfL. However, future studies with larger samples are needed to replicate these findings. Our sample size also limited our ability to match decline and stable groups by sex, and although groups generally showed no significant differences, sex‐dependent variation has been reported in AD progression.^42^, ^43^, ^44^, ^45^

Our findings highlight the necessity of BMI‐ and BV‐informed BBM models to enhance the accuracy of amyloid PET prediction and early AD detection and suggest that failing to account for BMI and BV could obscure pathological changes in asymptomatic individuals. The association between declining BMI/BV and increased BBM variability suggests that weight loss—whether due to aging, lifestyle, or GLP‐1 receptor agonist use—may impact BBM reliability to predict brain pathology. Moreover, failing to adjust for longitudinal changes in these measures may lead to underestimation of ATN/IVS classification in participants who experiences increases, and overestimation in those who experience decline.

Future studies should consider additional factors that can influence BV, which were not available for our analysis. These include hydration, pregnancy, lifestyle factors like physical activity, medications, and conditions such as chronic kidney disease, congestive heart failure, liver disease, and anemia. Another limitation of our analysis was the estimation of BV using the Nadler formula, which relies on height and weight. Future studies should consider more precise BV quantification, such as radiolabeled tracer dilution methods. These considerations may be particularly meaningful when investigating these effects in cognitively impaired populations, given that BBMs currently approved for diagnostic use are primarily centered around individuals with MCI. Finally, our study did not include tau PET or structural MRI, which could provide further insight into the impact of blood dilution on BBMs, especially p‐Tau_217_ and NfL, within the greater ATN/IVS framework. Expanding these investigations with larger, more diverse and obesity‐enriched cohorts, precise BV quantification, and multimodal imaging could further refine our understanding of how physiological factors impact BBM accuracy in AD diagnostics.

## 5 CONCLUSION

This study highlights the impact of BMI and BV on BBMs, particularly p‐Tau_217_ and NfL, due to dilution effects. While BMI and BV generally did not associate with amyloid PET directly, they moderated the relationships between BBMs and PET, attenuating expected associations through dilution. Stratifying BBM cutoffs by BMI improved amyloid PET classification, reducing misclassification rates. Additionally, BMI and BV declines introduced variability, leading to systematic overestimation of PET SUVR based on BBM concentrations. Given the growing clinical reliance on BBMs, BMI‐ and BV‐adjusted models and stratified thresholds might be critical for improving diagnostic accuracy and early detection of AD pathologies.

## CONSENT STATEMENT

All ADNI participants provided informed consent.

## CONFLICT OF INTEREST STATEMENT

J.F. reported serving on the advisory boards, adjudication committees, or speaker honoraria from AC Immune, Adamed, Alzheon, Biogen, Eisai, Esteve, Fujirebio, Ionis, Laboratorios Carnot, Life Molecular Imaging, Lilly, Lundbeck, Novo Nordisk, Perha, Roche, Zambón, Spanish Neurological Society, T21 Research Society, Lumind foundation, Jérôme‐Lejeune Foundation, Alzheimer's Association, National Institutes of Health USA, and Instituto de Salud Carlos III. J.F. reports holding a patent for markers of synaptopathy in neurodegenerative disease (licensed to ADx, EPI8382175.0). M.M.M. has served on scientific advisory boards and/or has consulted for Acadia, Beckman Coulter, Biogen, Cognito Therapuetics, Eisai, Lilly, Merck, Neurogen Biomarking, Novo Nordisk, Roche, Siemens Healthineers. She receives funding from the Alzheimer's Association, Davos Alzheimer's Collaborative, Department of Defense and National Institutes of Health. T.M.S. serves as an Equity & Scientific advisor for MICroStructure Imaging. The remaining authors have declared no conflicts of interest. Author disclosures are available in the Supporting Information.

## ETHICS APPROVAL AND CONSENT TO PARTICIPATE

Each site involved in data collection for the ADNI received local Institutional Review Board (IRB) approval. Written informed consent was obtained from each enrolled participant.

## Supporting information



Supporting Information

Supporting Information

## Data Availability

All data and code used in this analysis are available to ADNI‐approved, qualified investigators upon request.
